# La grossesse ovarienne à propos d’un cas et revue de la littérature

**DOI:** 10.11604/pamj.2021.40.208.32011

**Published:** 2021-12-07

**Authors:** Olfa Zoukar, Ines Zouari, Yosra Jemaa, Rahma Aissa, Amina Mnejja, Amel Bayar, Dalel Naguez, Dhekra Toumi, Mossaab Ghannouchi, Anis Haddad

**Affiliations:** 1 Centre de Maternité et Néonatologie, Centre Hospitalier Universitaire Fattouma Bourguiba de Monastir, Monastir, Tunisie,; 2 Service de Gynécologie Obstétrique, Hôpital Régional Hadj Ali Soua, Ksar Hellal, Tunisie,; 3 Service de Gynécologie Obstétrique du Centre Hospitalier Universitaire Taher Sfar, Mahdia, Tunisie,; 4 Service de Chirurgie Générale du Centre Hospitalier Universitaire Taher Sfar, Mahdia, Tunisie

**Keywords:** Grossesse ovarienne, ectopie rare, échographie, traitement chirurgical, cas clinique, Ovarian pregnancy, rare form of ectopic pregnancy, ultrasound, surgical treatment, case report

## Abstract

La grossesse ovarienne représente une entité rare parmi les grossesses ectopiques. Son diagnostic et sa prise en charge ne sont pas toujours aisés. C´est une pathologie particulière, le clinicien est confronté à une sémiologie clinique pauvre et à un diagnostic échographique difficile. Les critères chirurgicaux restent difficiles à prouver. Nous avons colligé un cas de grossesse ovarienne. La patiente a consulté nos urgences pour des douleurs pelviennes, métrorragies et une aménorrhée de 9 semaines d’aménorrhée (SA). Le diagnostic préopératoire a été évoqué à l´échographie qui a montré une image latéro utérine droite de 7*8cm au dépend de l’ovaire droit. Une laparotomie a été réalisée en urgence. Le traitement chirurgical a été radical après l´échec du traitement conservateur. La grossesse ovarienne est une entité rare de la grossesse extra-utérine qui présente certaines particularités sémiologiques. Son diagnostic est difficile et se base sur des constatations per-opératoires. Sa prise en charge thérapeutique reste pour le traitement des grossesses extra-utérines, malgré le progrès de traitement médical, chirurgicale.

## Abstract (en)

Ovarian pregnancy is a rare entity among ectopic pregnancies. Its diagnosis and management are not always easy. It is a particular pathology, the clinician is confronted with a poor clinical semiology and a difficult ultrasound diagnosis. The surgical criteria remain difficult to prove. We have compiled a case of ovarian pregnancy. The patient consulted our emergency room for pelvic pain, metrorrhagia and amenorrhea of nine weeks. The preoperative diagnosis was evoked by ultrasound which showed a right latero uterine image of 7*8cm at the expense of the right ovary. An emergency laparotomy was performed. Surgical treatment was radical after the failure of conservative treatment. Ovarian pregnancy is a rare entity of ectopic pregnancy which presents certain semiological peculiarities. Its diagnosis is difficult and is based on intraoperative findings. Its therapeutic management remains for the treatment of ectopic pregnancies, despite the progress of medical and surgical treatment.

## Introduction

La grossesse extra-utérine (GEU) est l´une des urgences médicochirurgicales les plus fréquentes en gynécologie. La grossesse ovarienne (GO) est une variété de grossesse où l'ovaire est le siège de la nidation [1]. Elle occupe une place particulière parmi les grossesses ectopiques en raison de sa rareté qui est liée d'une part à sa définition qui prend en compte des critères anatomiques, et d'autre part à des démarches diagnostiques bien codifiées. La grossesse ovarienne est l'une des rares grossesses extra utérines avec un diagnostic souvent réalisé lors des interventions [1]. Contrairement, aux autres types de grossesse extra-utérine (GEU), la GO reste un phénomène isolé et exceptionnel, indépendant des facteurs de risques habituels. D'autant plus que le mécanisme exact aboutissant à une GO est encore mal élucidé. Par rapport aux autres GEU, d'autres formes de révélation de la GO ont été rapportées comme quoi la GO peut évoluer jusqu'au 2^e^ trimestre, voire jusqu'à terme.

## Patient et observation

Il s´agit d´un cas de grossesse extra-utérine, ovarienne observé au Centre de maternité et de néonatologie de Monastir (CMNM). Les données sont recueillies à partir du dossier médical. Le diagnostic a été évoqué devant des données biologiques, échographiques, des constatations per-opératoires. Les données de l´examen anatomopathologique ont permis de confirmer le diagnostic. La prise en charge thérapeutique a été chirurgicale en urgence.

### Présentation du cas clinique

**Informations relatives aux patients:** madame AR âgée de 43 ans, GS O positif, consulte nos urgences pour des douleurs pelviennes, métrorragies et une aménorrhée de 9 SA. Elle a dans ses antécédents cardiovasculaires (ATCD) un diabète sous ado. C´est une G7P5A1, 5AVB, avec une grossesse arrêtée à la 7^e^ semaine d’aménorrhée (SA) curetée. Pas de notion de prise de pilule, contraception par préservatif, opérée pour une appendicite aiguë il y a de cela 5 ans.

**Résultats cliniques:** l´examen à l´admission a trouvé des conjonctives très pâles, TA à 9/6 et pouls à 120 bpm, une sensibilité abdominale diffuse surtout exagérée au niveau de la fosse iliaque droite. Une cicatrice pelvienne de Mac Burney. L´examen au spéculum a objectivé un saignement noirâtre de faible abondance d´origine end utérin avec une douleur à la mobilisation utérine surtout à droite.

**Chronologie:** les douleurs pelviennes et les métrorragies sont installées depuis un jour de son admission avec notion d´aggravation des douleurs pelviennes ramenant la patiente à consulter d´urgence.

**Démarche diagnostique:** l´échographie endovaginale a objectivé un utérus vide, endomètre fin, avec la présence d´une masse latéro utérine droite faisant 8X7cm, hétérogène, échogène aux dépens de l´ovaire droit, avec la présence d´un épanchement de grande abondance. Le taux de BHCG est revenu positif à 73475mUI/ml, HB à 7,8g/dl, TP à 80%. Le diagnostic préopératoire le plus probable est la grossesse extra-utérine ovarienne rompue, compliqué d'un état de choc hémorragique, avec pronostic vital mis en jeu, la prise en charge chirurgicale doit être très rapide.

**Figure 1:**
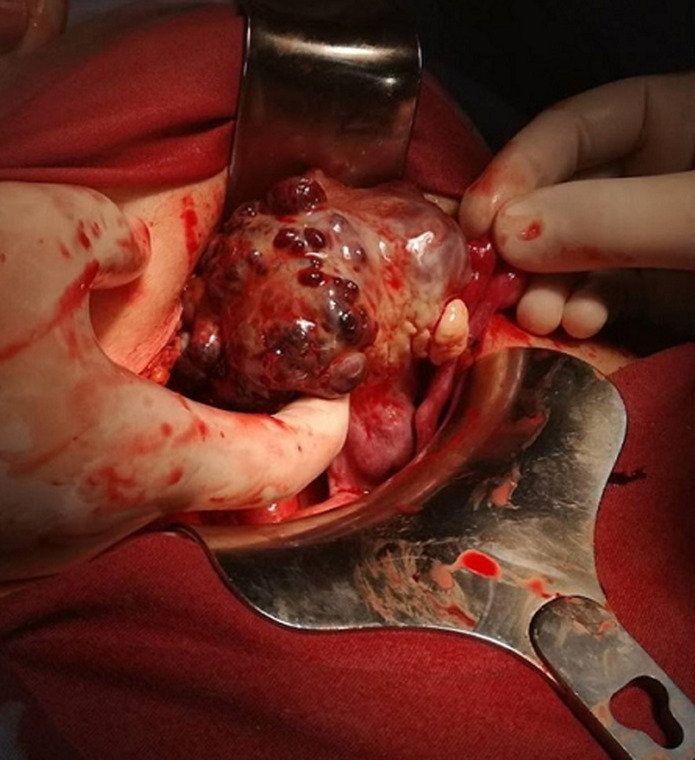
grossesse extra utérine ovarienne en per opératoire

**Figure 2:**
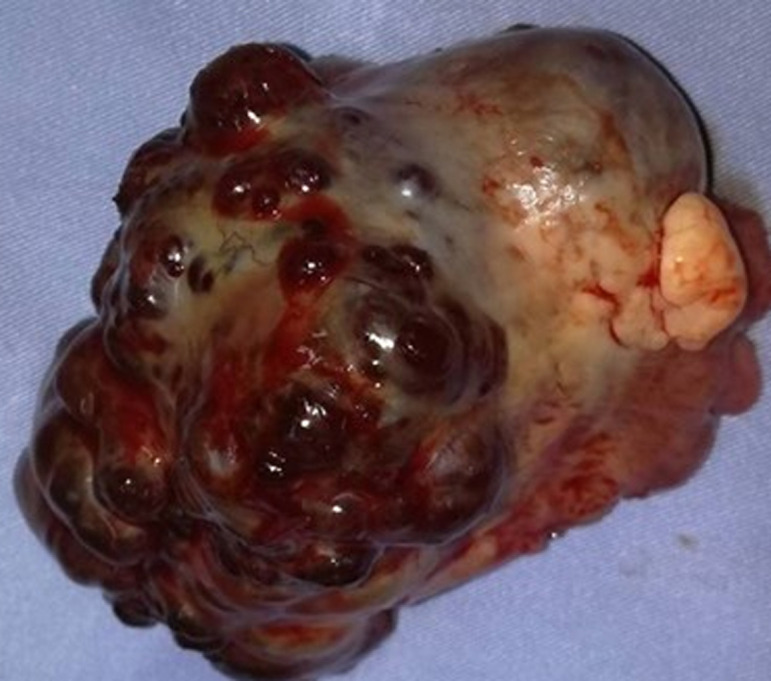
ovairectomie droite

**Intervention thérapeutique:** une laparoscopie a été pratiquée en urgence pour suspicion d´une grossesse extra-utérine très probablement ovarienne selon les données de l´échographie. L´exploration peropératoire a montré la présence d´un épanchement de grande abondance fait du sang caillouté faisant environ 800cc, utérus de taille et de contours régulier, annexes gauches sans particularité, l´annexe droit: présence d´une masse faisant environ 10 x 9,5cm de grand axe aux dépens de l´ovaire droit avec un saignement actif au niveau de la masse (Figure 1). Il s´agit d´une grossesse extra-utérine ovarienne droite rompue avec un saignement actif. L´excision du trophoblaste a été difficile et hémorragique, justifiant le recours à une résection partielle de l´ovaire pour assurer l´hémostase, mais devant le saignement actif au niveau de l´ovaire droit et l´hémostase qui reste incontrôlable, on a décidé de faire une ovariectomie droite associée à une salpingectomie droite avec une ligature de la trompe gauche selon le désir et le consentement de la patiente avant de commencer l´acte chirurgicale (Figure 2).

**Suivi et résultats:** les suites postopératoires étaient simples. L´examen anatomopathologique a confirmé le diagnostic de grossesse ovarienne.

**Perspective du patiente:** la patiente a exprimé son avis à propos du traitement chirurgical de la grossesse ovarienne en indiquant son consentement pour ce traitement radical.

## Discussion

La grossesse ovarienne (GO) représente 3% des grossesses extra-utérines [2]. Sa physiopathologie est mal connue, elle semblerait être secondaire à un reflux de l´ovocyte fécondé vers l´ovaire [2]. Les cas de GO après fécondations in vitro rapportées par la littérature confortent la théorie du reflux [3]. La grossesse s´implante préférentiellement sur la cicatrice de l´ostium folliculaire d´origine, riche en fibrine et en néo capillaires [4]. Cette théorie correspond aux formes intra folliculaire et juxta folliculaire. Plus rarement, cette implantation va se faire à distance du corps jaune ou même sur l´ovaire controlatéral, correspondant alors aux formes juxta corticale et interstitielle dont la physiopathologie demeure obscure. Plus rarement, la GO peut être bilatérale ou faire partie d´une grossesse hétérotopique [5]. Dans notre série, toutes les grossesses étaient uniques et implantées du côté de leur corps jaune. Contrairement aux GEU tubaires, la pathologie et la chirurgie tubaire ne semblent pas augmenter le risque de survenue de GO. Cependant l´incrimination des pathologies inflammatoires du pelvis dans la genèse des GO ne fait pas l´unanimité des auteurs [6]. Par ailleurs, la contraception par un dispositif intra utérin paraît particulièrement associée aux grossesses ovariennes [7]. En effet, plusieurs auteurs sur des séries de 7 à 26 GO [8] ont noté des proportions allant de 57 à 90% de patientes porteuses de stérilet. Cliniquement, la symptomatologie douloureuse abdominopelvienne devance la scène. Ces douleurs correspondent à la rupture de la capsule ovarienne par la grossesse et à la constitution de l´hémopéritoine [9]. Les patientes sont le plus souvent vues dans un contexte d´urgence, en état de choc [10].

Chez nos patientes, la symptomatologie douloureuse abdominopelvienne était effectivement au premier plan, et une patiente était en état de choc hémorragique. Le diagnostic de grossesse ovarienne peut être évoqué à l´échographie par un opérateur performant. On peut mettre en évidence un sac gestationnel attenant à l´ovaire ou comme certains l´on décrit, un double anneau hyperéchogène au sein d´une masse latéro-utérine hypoéchogène avec ou sans embryon [11]. En effet, selon l´âge de la grossesse, plusieurs images échographiques ont été décrites dans la littérature [12]. Certains critères échographiques sont très suggestifs de la localisation ovarienne de la grossesse: la présence d´image ronde anéchogène avec une couronne hyperéchogène à la surface de l´ovaire, la présence de parenchyme ovarien comme un corps jaune ou un follicule entourant la masse, et une echogènicité de la masse plus élevée que celle de l´ovaire [13]. Le diagnostic différentiel se pose souvent avec un kyste du corps jaune ou un kyste hémorragique. Dans ce cas, l´échographie tridimensionnelle (3D) semble pouvoir faire la différence grâce aux plans de coupe [14]. Le Doppler énergie ne semble pas intéressant pour le diagnostic [15]. Le Doppler pulsé semble avoir plus d´intérêt. Plusieurs techniques chirurgicales ont été décrites: résection cunéiforme de l´ovaire emportant la GO, énucléation de la GO, kystectomie du corps jaune emportant le trophoblaste, curetage du trophoblaste avec coagulation ou surjet hémostatique du lit de la GO avec conservation totale de l´ovaire [16].

Dans de rares cas, du fait du développement avancé de la grossesse, l´ovariectomie voire l´annexectomie peut s´imposer [12]. C´est le cas de notre patiente. En effet, la GO est souvent diagnostiquée au stade de complications empêchant le recours au traitement médical en première intention [11]. L´adjonction de méthotrexate (MTX) peut s´envisager en rattrapage d´un traitement chirurgical insuffisant. Nous n´avons pas eu recours au MTX. Concernant son pronostic, la GO, du fait de l´absence d´atteinte tubaire, ne constitue pas un facteur de risque d´une nouvelle GEU [1]. Un seul cas de récidive de GO a été décrit dans la littérature et a concerné l´ovaire controlatéral [10]. L'examen anatomo-pathologique revêt une importance capitale car c'est lui qui permet de confirmer le diagnostic de GO. Il a pour but d'éliminer les grossesses abdominales primitives, celles greffées sur l'ovaire mais provenant d'un avortement tubo-abdominal, et celles où l'ovaire n'est pas le siège exclusif de la nidation, d'après les critères anatomiques de Spielberg en 1878 [13] la trompe du côté atteint, y compris le pavillon, doit être indemne de toute lésion; le sac ovulaire doit occuper la place anatomique habituelle de l'ovaire; l'ovaire et le sac gestationnel doivent être reliés à l'utérus par le ligament utéro-ovarien; il doit exister du tissu ovarien au sein du sac ovulaire 'ce qui sous-entend la confirmation histologique de la présence de villosités choriales au sein du tissu ovarien. A partir des critères anatomiques définis par certains auteurs [14,15], plusieurs classifications de GO ont été proposées.

## Conclusion

La grossesse ovarienne est une pathologie rare qui présente certaines particularités sémiologiques par rapport aux autres GEU. Son diagnostic est difficile. Sa prise en charge thérapeutique reste chirurgicale. La grossesse ovarienne bien que rare, demeure une urgence obstétricale avec une séméiologie réservée et particulière dépendant des complications selon les périodes dans l'évolution de la grossesse. Son diagnostic reste difficile et se fait souvent en peropératoire, à travers l´échographie par une main expérimentée au vu des possibilités faibles que peut présenter la séméiologie et repose aussi sur des constatations per- opératoires. La prise en charge est chirurgicale malgré les progrès du traitement médical.

**Un consentement** éclairé pour la publication de leurs détails cliniques et/ou images cliniques a été obtenu du patient.
